# Age-related changes in molecular organization of type I collagen in tendon as probed by polarized SHG and Raman microspectroscopy

**DOI:** 10.1038/s41598-019-43636-2

**Published:** 2019-05-13

**Authors:** Laurence Van Gulick, Charles Saby, Hamid Morjani, Abdelilah Beljebbar

**Affiliations:** BioSpectroscopie Translationnelle (BioSpecT), EA 7506, SFR CAP-Sante FED4231, Université de Reims Champagne-Ardenne, UFR de Pharmacie, 51 rue Cognacq-Jay, 51096 Reims, cedex France

**Keywords:** Raman spectroscopy, Diagnostic markers

## Abstract

Type I Collagen is one of the most abundant proteins of the extracellular matrix of the most organs. During chronological aging or in diseases, type I collagen undergoes biochemical and structural changes which can impact biomechanical and physiological properties of organs. In this study, we have investigated the age-related changes in the molecular organization of type I collagen in rat tails tendon using polarized Raman spectroscopy. Our results show that Amide I, amide III as well as the bands related to proline and hydroxyproline are highly sensitive to polarization and age-related. On the other hand, 1453 and 1270 cm^−1^ do not show any preferential orientation. Depolarization and anisotropic ratios were used to provide information about the changes in orientation of collagen fibers with aging. The anisotropy degree of Raman bands increase from adult to old collagen, indicating a higher collagen fibers alignment to the fascicle backbone axis in old tendons, and consequently a higher straightness of collagen fibers. These data were correlated to those obtained using polarized second harmonic generation technique. Polarized Raman mapping showed a more homogeneous spatial distribution of collagen fibers alignment to the fascicle axis in old tendon. This confirms a higher straightness of collagen fiber with aging.

## Introduction

**Figure 1:**
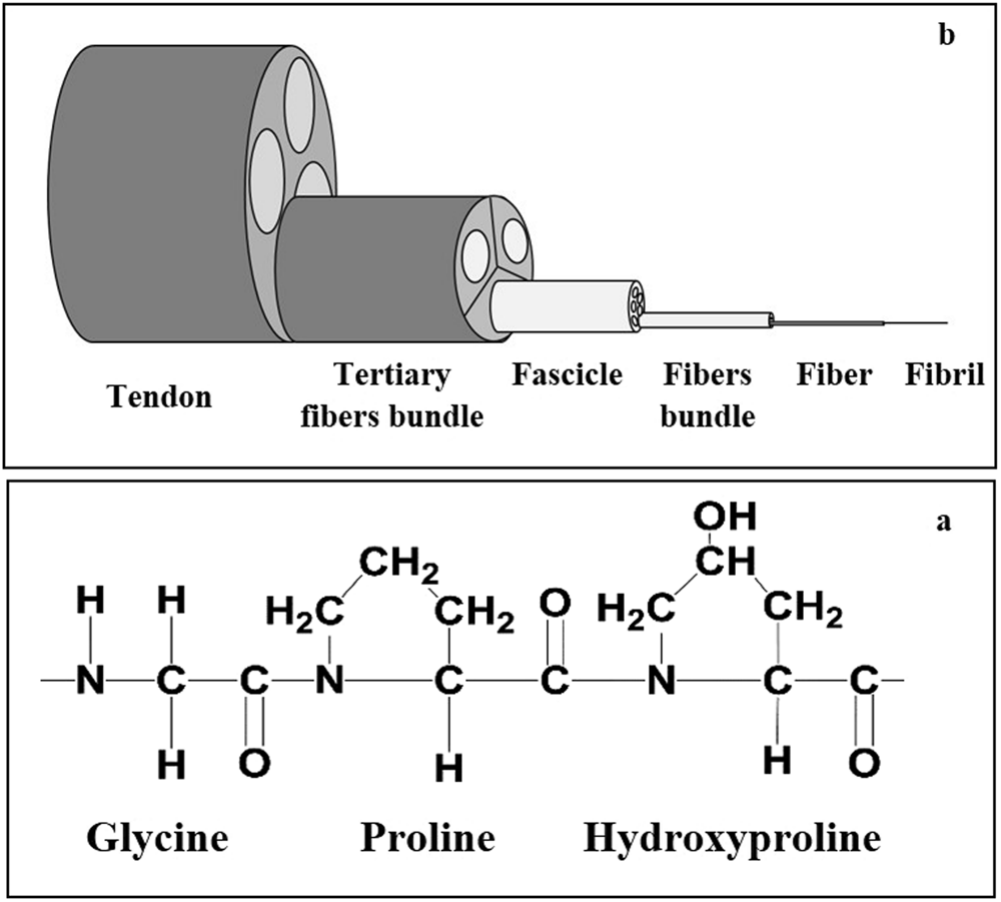
(**a**) Typical representation of 2 D sequence of type I collagen with major amino acid components Gly-Pro-HyPro and (**b**) Schematic representation of the structural hierarchy in RTT showing the relationship between fibrils, fibers, fiber bundle, fascicles and tendon unit.

Type I collagen is a major component of extracellular matrix (ECM) in several tissues. Its structural organization plays a crucial role in biological events such as cell migration, adhesion, and proliferation^1^. The primary structure of type I collagen corresponds to amino acid motif (Gly-X-Y) repeat, where X and Y are frequently represented by proline (Pro) or hydroxyproline (HyPro) amino acids respectively (Fig. 1a). The protein is organized as triple helical chains, assembled into fibrils, and stabilized by the formation of intermolecular and interfibrillar cross-links. As example, the main constituent of the rat tail tendon (RTT) is type I collagen (~90% of dry weight). Collagen fibers are organized in hierarchical manner and aligned in parallel to the tail (Fig. 1b)^2^. During chronological aging and in diseases, type I collagen undergoes several non-enzymatic post-translational modifications such as glycation. This process induces the generation of advanced glycated products (AGEs), which contribute to an increase in collagen fibers cross-links. This decreases also the individual collagen fiber diameter and length^3–9^. Several techniques have been used to provide detailed information on the organization of collagen fibers in tissues at *in vitro* and *in vivo* levels^10–13^. Polarized second harmonic generation (pSHG) is an emerging powerful technique to investigate morphological and organization of collagen fibers in tissues. This nonlinear optical technique is highly sensitive to changes in the structural organization of collagen that occurs in tissues during normal and pathological tissue transformation^14^.

Polarized Raman spectroscopy (PRS) is a new emerging approach to investigate specifically, the organization of type I collagen fibers at the molecular level^15–20^. This technique is able to bring very useful information on the anisotropic response of chemical bonds using different polarization directions for excitation and scattered lights. In, addition, Raman spectroscopy is non-destructive technique that provides chemical composition of the sample with a high spatial resolution without any labeling (label-free). By using the polarization properties of light (excitation and collection) and imaging, it is possible to access to the orientation of spatially organized molecules such as type I collagen. As mentioned above, aging is accompanied by changes in molecular organization of type I collagen, and consequently the biomechanical properties. Several studies have used PRS to characterize the anisotropic response of collagen in tendon^15,16^, to investigate the structure and orientation of collagen fibers in tendon and bone^17,18^, to quantify the degradation of collagen^19^, to discriminate between healthy and pathological tissues^20^.

In this study, we have investigated the age-related changes in the molecular organization, and orientation of collagen in hydrated RTTs using PRS and pSHG techniques. For PRS, tail tendons from adult (2 months) and old (24 months) rats were analyzed in hydrated conditions by combining different polarization directions of incident laser and collected Raman scattering. We aimed in this study i) to identify the highly anisotropic Raman bands of collagen bonds, ii) to find the polarization directions that bring spectral information related to the structural organization of collagen fibers, and iii) to identify those which could be affected by chronological aging.

## Results

**Figure 2:**
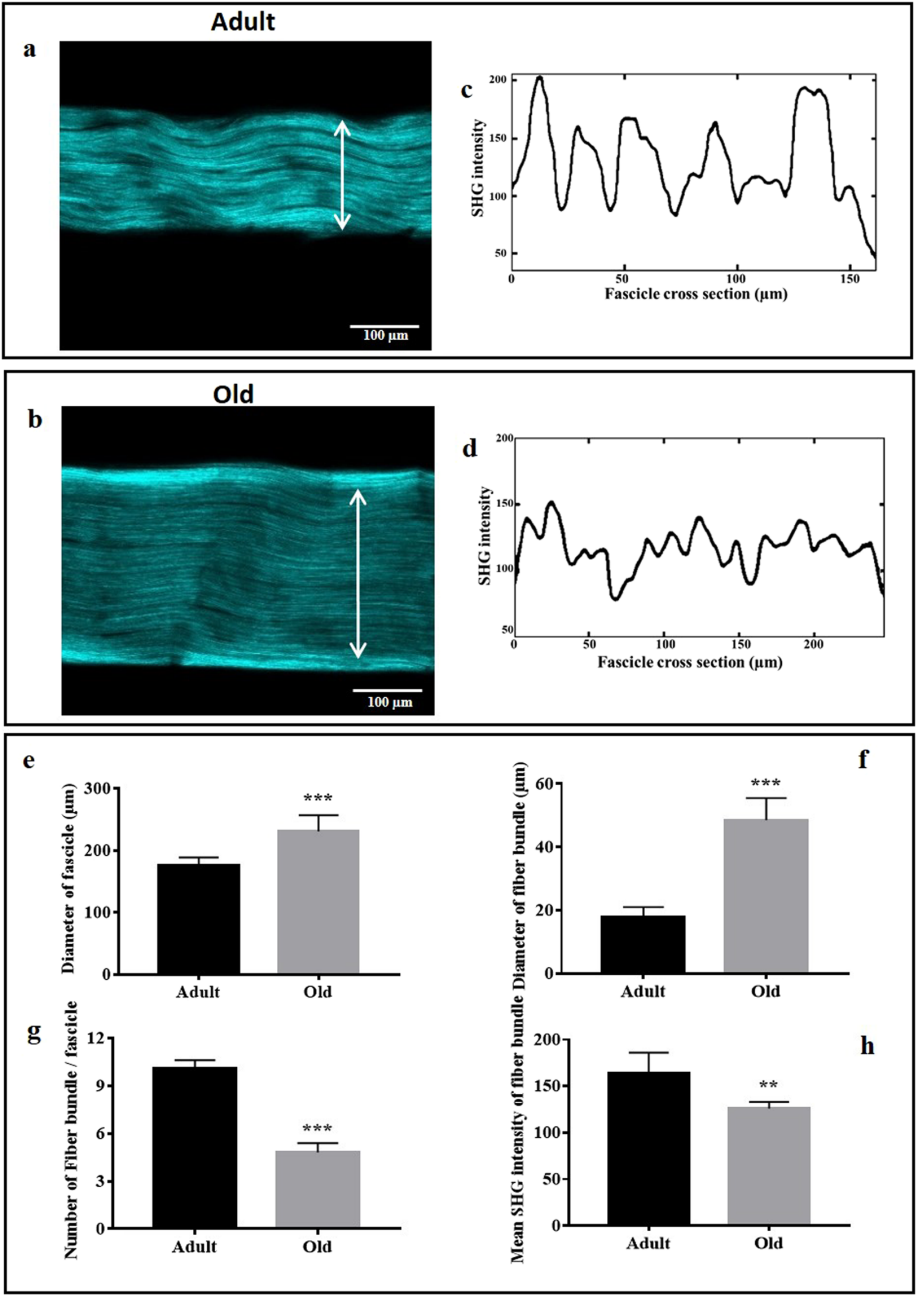
Polarized SHG images acquired on adult (**a**) and old (**b**) in fully hydrated RTT conditions up to the scale of collagen fiber bundles. Morphological changes are visible in the SHG signal. Fascicle cross section profile plots corresponding to the pixel intensity values along the selected vertical lines from the SHG images of adult (**c**) and old (**d**) RTT. The x-axis represents distance along the line (in µm) and the y-axis is the pixel intensity. The histograms (**e**) and (**f**) displayed the mean diameters of collagen fascicles and, fiber bundles respectively; (**g**) and (**h**) showed respectively the number of fiber bundles and the mean SHG intensity of fiber bundles per fascicle measured on several adult and old RTTs.

pSHG imaging technique was used to investigate the morphological organization of fascicle and fiber bundles on hydrated adult and old RTTs. Figure 2a,b display a fibrillar alignment of the collagen in adult and old RTTs. Data shows the age-dependent differences in the fibrillar organization. Indeed, pSHG image of adult RTTs exhibits fascicles organized as periodic waveform configuration with a long axis parallel to the fascicle backbone axis (Fig. 2a), indicating a low straightness of collagen fibers. This organization disappears in old RTTs (Fig. 2b). In fact, the fibers become linear and oriented in a parallel manner to backbone axis. This indicates a high straightness of collagen fibers in old RTTs (data not shown). The average diameters of fascicles in adult and old tendons were then measured. The mean fascicle diameter values were 176 ± 13 µm and 231 ± 26 µm for adult and old RTTs respectively (Fig. 2e). In addition, it was possible to observe several dark regions in adult tendon due to inter fascicle spaces. The number of these dark regions decreases in the old tendons. These inter-fascicle spaces were used to measure the mean diameters of adult and old fiber bundles. The fascicle cross section profile plots were generated to estimate the mean diameters of fiber bundles based on width of the periodic waveform (Fig. 2c,d). The mean diameters of fiber bundles for adult and old RTTs were 18 ± 4 µm and 48 ± 20 µm respectively (Fig. 2f). Bundle fiber number was calculated in adult and old fascicles. As shown in Fig. 2g, mean bundle fiber number was significantly higher in adult (10.1 ± 0.5) than in old RTTs (4.8 ± 0.6) fascicle. This decrease in bundle fiber number with aging suggests a lower collagen density in old fascicles. In order to determine whether collagen density was impacted by aging, SHG intensity of fiber bundle has been measured. As shown in Fig. 2h, SHG intensity was significantly decreased from adult collagen (164 ± 22) to old collagen (126 ± 7).

**Figure 3:**
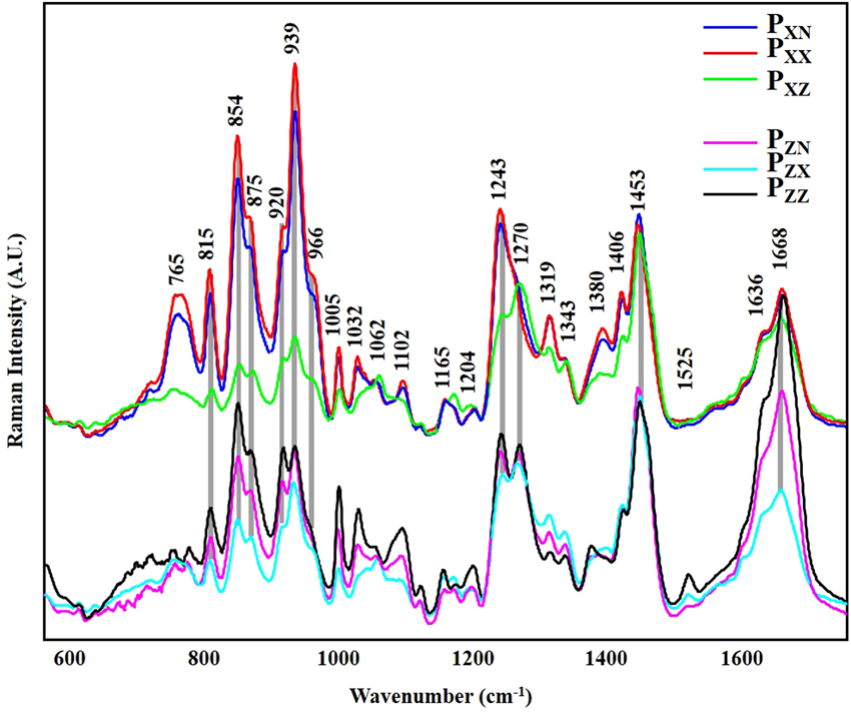
Conventional (P_XN_ and P_ZN_) and polarized (P_XX_, P_XZ_, P_ZX_, P_ZZ_) averaged Raman spectra measured from the same areas on fresh adult RTTs by combining different polarization directions of the incident laser and Raman collections. Spectra were grouped according to horizontal (X) or vertical (Z) laser directions. Each mean spectrum was obtained by averaging several hundred spectra, recorded with integration time of 20 s. The spectra were normalized on the intensity of the band at 1453 cm^−1^.

The anisotropic properties of Raman signal from collagen fibers of adult and old RTTs have been investigated using polarized Raman (PR) microspectroscopy. These properties allowed the identification of privileged orientation of collagen bonds with respect to the fascicle backbone axis (incident and collected light polarization directions). Conventional (P_XN_ and P_ZN_) and polarized (P_XX_, P_XZ_, P_ZX_, and P_ZZ_) Raman spectra were recorded from the same areas of hydrated adult and old RTTs. The spectra are presented according to the polarization of incident laser (X or Z directions) (Fig. 3). These spectra were normalized with respect to the isotropic band at 1454 cm^−1^ (see materials and methods). Conventional Raman spectrum (P_XN_) exhibits the main characteristic bands of type I collagen^21^. Table 1 listed the frequencies and tentative Raman bands assignments of type I collagen. The amide I band is composed of two vibration frequencies located at 1636 and 1668 cm^−1^ which are attributed to the carbonyl groups of proline and hydroxyproline respectively. The amide III bands at 1270 and 1243 cm^−1^ arise from N–H bending and C–N stretching^22^.

**Table 1:** Raman bands assignment of type I collagen in the 600–1800 cm^−1^ spectral region.

Band frequency (cm^−1^)	Assignment
1668	Amide I ν(C=O)
1636	Amide I ν(C=O)
1453	δ(CH_2_, CH_3_),
1319	γt(CH2)
1270	Amide III δ(NH_2_)
1243	Amide III (C-N)
1003	Phe
938	ν(C-C) of protein backbone
920	ν(C-C) of Pro ring
875	ν(C-C) of Hyp ring
854	ν(C-C) of Pro ring
815	ν(C-C) of protein backbone
765	δCOO-

The comparison between conventional and polarized Raman spectra of adult RTTs shows differences in the relative intensities of several bands (Fig. 3). The intensity of the band at 1668 cm^−1^ reaches the maximal value in P_ZZ,_ while it was highly decreased in P_XX_. However, this band did not completely disappear in P_XX_, indicating that the C=O bonds are oriented almost but not completely perpendicular to the direction of the fibril axis (see Fig. 1a). The intensity ratio 1668/1636 cm^−1^ did not change when P_XN_ and P_XX_ were used. However, this ratio increases with vertical laser polarization (P_ZZ_ and P_ZN_). This could be explained by the moderate vertical orientation of the carbonyl groups of proline when compared to hydroxyproline. The sensitivity of amide III band to polarization was completely different from that on amide I band. In fact, data showed that C-N bonds are oriented both in perpendicular (1270 cm^−1^) and parallel (1243 cm^−1^) directions to the fascicle backbone axis (Fig. 1a). The intensity of the band at 1270 cm^−1^ is similar between horizontal laser polarization (P_XX_ and P_XZ_) and vertical laser direction (P_ZX_ and P_ZZ_). This suggests that this vibration is not sensitive to polarization. The sensitivity of 1243 cm^−1^ band is quite different from that at 1270 cm^−1^. In fact, the intensity of this band is very high in P_XX_ and P_XN_ directions and decreases in P_ZN_, P_ZZ_, P_ZX_ meaning that this vibration is assigned mainly to parallel C-N bonds. The band at 939 cm^−1^ (C–C vibration parallel to the fascicle backbone axis) is one of the most sensitive to polarization. This band presents high intensity in P_XN_ and P_XX_, which decreases in P_ZZ_ and P_ZX_ and P_XZ_. The sensitivity of the 815 cm^−1^ band (C–C skeletal stretching) is similar to that at 939 cm^−1^. The relative intensities of the bands at 920 cm^−1^ (C–C stretch Pro ring of collagen) and 854 cm^−1^ and 875 cm^−1^ (C-C stretching of proline and hydroxyproline rings respectively) are higher in P_XN_ and P_XX_ than in P_ZN_ and P_ZZ_. In the case of crossed-polarization directions P_ZX_ or P_XZ_, the intensities of these bands decreased significantly. This suggests that the associated bonds possess parallel and perpendicular vibrations components. The band at 765 cm^−1^ (C-CO) presented a higher intensity in P_XX_ and P_XN_ spectra when compared to P_ZZ_, P_ZX_, and P_XZ,_ suggesting that the bond corresponding to this vibration was parallel to the fascicle backbone axis. Finally, a band at 1525 cm^−1^, which does not correspond to collagen, was observed in spectra of adult RTTs only^23^. This band, which corresponds to C=C stretching vibration of carotenoids, decreases from P_ZZ_ to P_ZX_ in P_ZN_ directions and vanishes in P_XX_, P_XN_, and P_XZ_ directions.

**Figure 4:**
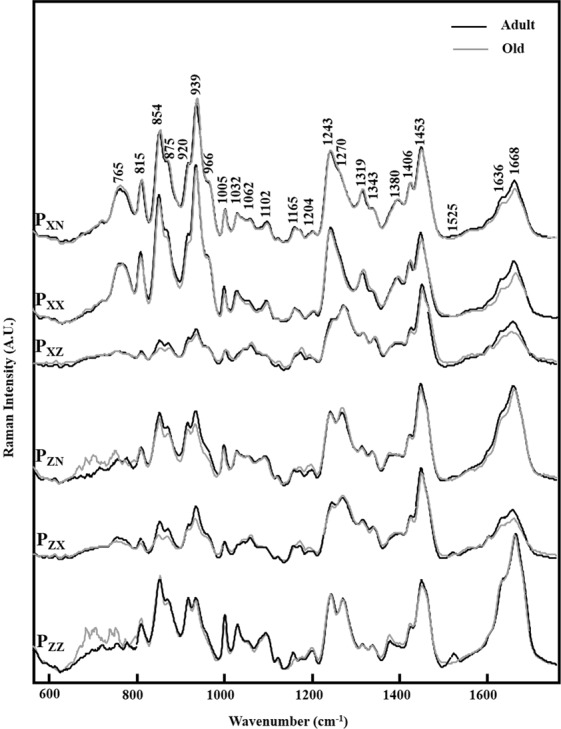
Mean conventional and PRS spectra measured on adult (black line) and old (grey line) RTT with the same experimental conditions. For each laser and Raman polarization direction, the spectra are compared according to the age with the aim to identify the changes in the relative intensities of the collagen bands between adult and old RTT samples.

The relationship between molecular organization of type I collagen and age-related changes has been then investigated. Figure 4 displays the comparison between Raman spectra recorded on adult and old RTTs using polarization properties, conventional (P_XN_ and P_ZN_) and polarized (P_XX_, P_XZ_, P_ZX_, and P_ZZ_) configuration. The spectra from old RTTs exhibit the main characteristic bands of type I collagen described above with changes in their relative intensity with aging specifically in the region of the amide I band and the proline/Hydroxyproline region 854–939 cm^−1^ (Fig. 4). In fact, in all polarization directions, the intensity of amide I band at 1668 cm^−1^ decreased in old RTTs except for P_ZZ._ The relative intensity of the bands related to Proline and Hydroxyproline vibrations (939, 920, 875, 854 cm^−1^) decreased in the case of old RTTs as compared to adult ones in P_XZ_, P_ZX_, and P_ZN_. In P_XX_, P_XN_, and P_ZZ_, the intensity of these bands did not change with aging (Fig. 4). Finally, the intensities of the bands at 765 cm^−1^ and 815 cm^−1^ are similar between adult and old RTTs in all polarization directions. All the differences presented above were correlated to the changes in the orientation in the type I collagen fibers with aging. In addition, the band at 1525 cm^−1^ observed in adult RTTs spectra completely disappeared in old ones.

**Figure 5:**
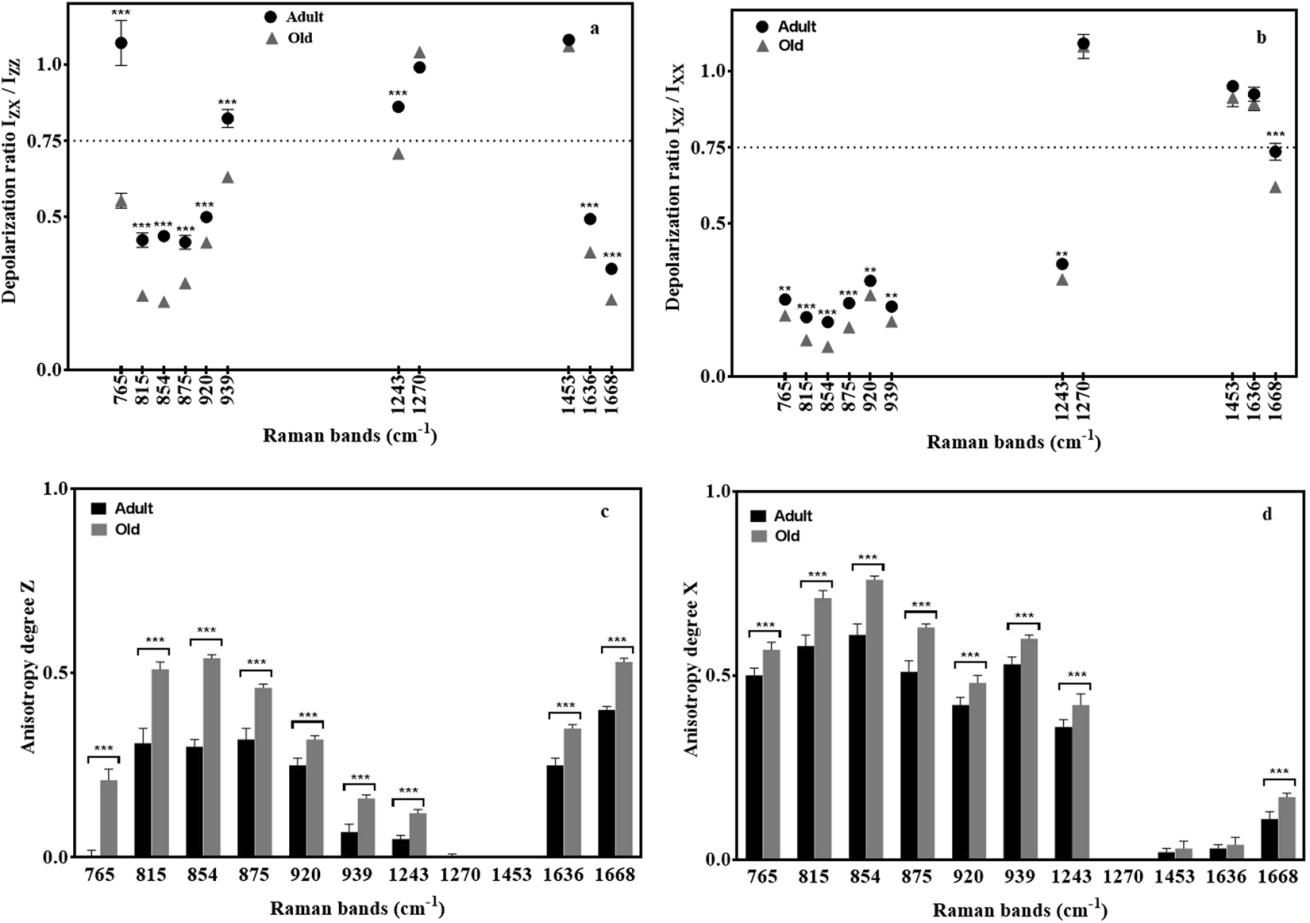
Depolarization ratios (ρ_z_) and (ρ_x_) (**a** and **b**) and polarization anisotropy (Az) and (Ax) (**c** and **d**) calculated on specific collagen bands from adult (black circle) and old (grey triangle). Mean +/−  standard deviation values are shown. Statistical analysis Anova test was used to determine whether there are any *statistically* significant differences between adult and old depolarization and anisotropic ratios (*p < 0.05, **p < 0.01, ***p < 0.001).

Depolarization ratio (ρ) was calculated to evaluate the sensitivity of collagen bands to polarization. This allowed to provide information on changes in collagen fiber alignment to the fascicle backbone axis and consequently in the fiber straightness with aging. Figure 5a,b display depolarization ratios (ρ_z_) and (ρ_x_) calculated from the relative intensity of the main characteristic bands of collagen of adult and old RTTs. High depolarization ratios ρ_Z_ and ρx (>0.75) were obtained for the CH_2_/CH_3_ (deformations) and amide III bands at 1453 and 1270 cm^−1^ respectively for adult and old collagen. This confirms that these bands are not sensitive to polarization. However, significant differences were observed between adult and old RTTs for ρ_Z_ of the other bands when compared to ρ_X_. In fact, these changes were more pronounced in P_ZZ_ and P_ZX_. For ρ_Z_ depolarization ratio, bands at 1243 and 939 cm^−1^ are considered as isotropic for adult collagen (ρ_Z_ >0.75) (Fig. 5a). These ratios decreased in old collagen (<0.75) indicating an increase in the degree of alignment of collagen fibers to the fascicle backbone axis with aging, and consequently and increase in the straightness of collagen fibers (Fig. 5a). On the other hand, the ρ_Z_ of the bands at 815, 854 875, 920, 1636, and 1668 cm^−1^ significantly decreased from adult to old collagen (p < 0.001). This suggests that the collagen fibers were highly aligned to the fascicle backbone axis in old RTTs than adult ones. ρ_Z_ of the band at 765 cm^−1^, corresponding to C-CO bond, decreased from 1 to 0.5 with aging. This indicates that this bond is preferentially oriented in the same direction as the fascicle backbone axis of old RTTs. Depolarization ratios ρx of the carbonyl bands at 1668 and 1636 cm^−1^ are around 0.75 in adult RTTs (Fig. 5b). No significant modification in the ρx of 1636 cm^−1^ band was observed between adult and old RTTs. However, the ρx of 1668 cm^−1^ band decreased significantly with aging (0.75 for adult and 0.65 for old collagen). The bands in the spectral region from 765 to 939 cm^−1^ were highly polarizable. However, ρx of bands at 815, 854, and 875 cm^−1^ decreased more significantly from adult to old collagen than those at 765, 920, and 939 cm^−1^.

The anisotropy degrees A_Z_ and A_X_ were calculated to determine the orientation of collagen fibers in adult and old RTTs (Fig. 5c,d). A_Z_ and A_X_ of the main bands were higher in old collagen when compared to adult one, indicating a higher alignment of collagen fibers to the fascicle backbone axis in old RTTs. However, these differences are less pronounced for Ax than for Az. This confirms that the most changes in the organization of collagen fibers are observed in the vertical direction. In fact, the predominant orientation of each collagen bond corresponds to the higher value between A_X_ and A_Z_. For example, the anisotropy degrees A_Z_ and A_X_ of the band at 1668 cm^−1^ were respectively 0.26 and 0.1 in adult and old RTTs. This suggests that C=O bonds are mostly oriented perpendicularly to the fascicle backbone axis. In the same way, A_X_ was higher than A_Z_ in the case of the proline and hydroxyproline bands (815, 854, 875, and 920 cm^−1^). This suggests that the bonds corresponding to these vibrations are mostly oriented in parallel direction to the fascicle backbone axis. A_Z_ and Ax of the bands at 1453 and 1270 cm^−1^ were almost equal to zero due to isotropic properties of these bands. For the band at 1243 cm^−1^, A_X_ value was higher than that of A_Z_ for both collagens. This indicates that the C-N bonds corresponding to this vibration is mostly oriented in parallel direction to the fascicle backbone axis. In addition, Ax was significantly lower in adult (0.36 ± 0.02) than in old collagen (0.42 ± 0.03) (p < 0.001).

**Figure 6:**
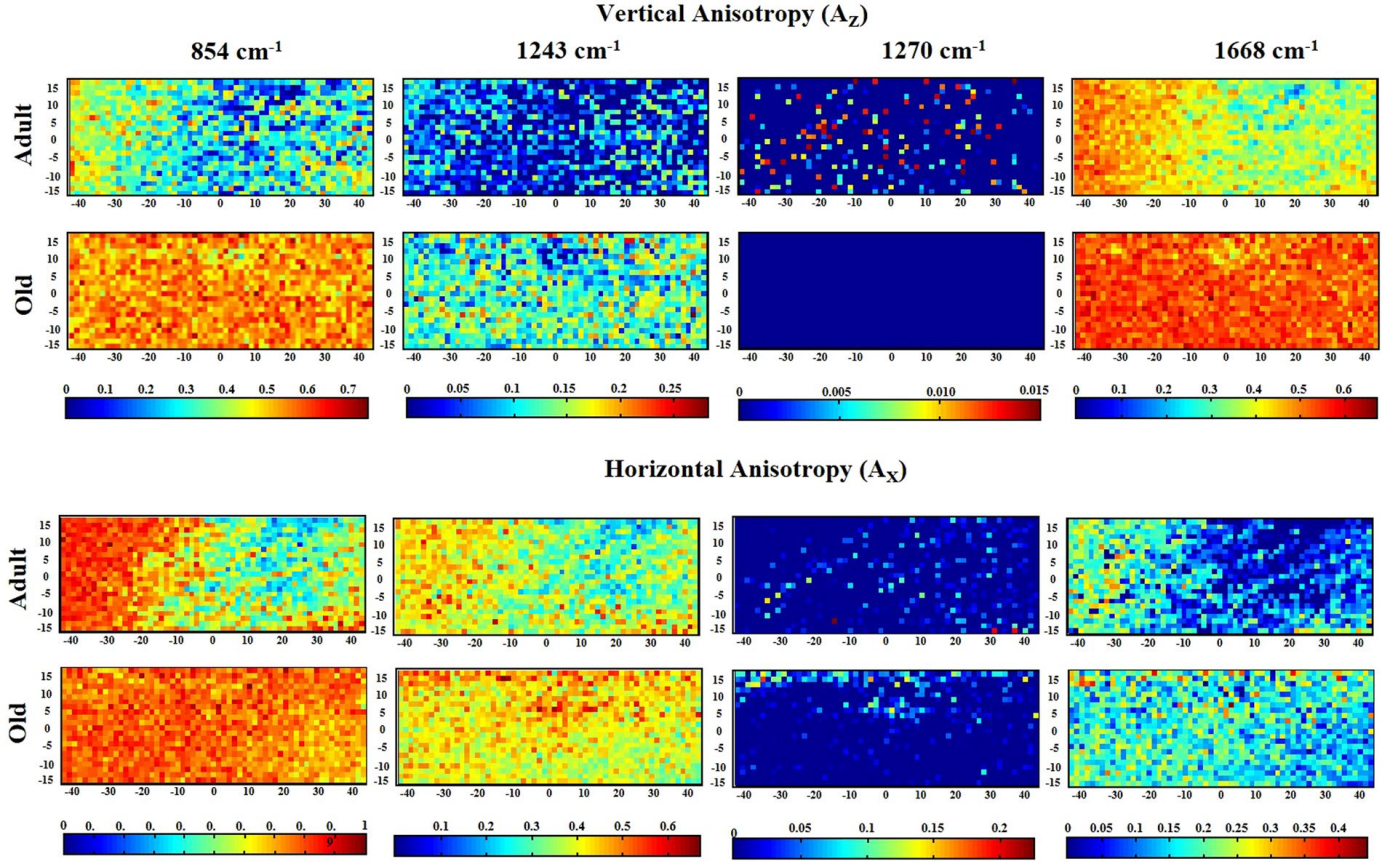
Maps representing the distribution of vertical and horizontal degree of anisotropy of the specific collagen band 1668, 1270, 1243, and 845 cm^−1^ from adult and old RTT were displayed. Significant changes were observed between adult and old collagen indicating a higher degree of molecular alignment occurs during chronological aging.

PR spectroscopy mapping was performed to evaluate the distribution of collagen fiber orientation in adult and old RTTs. Figure 6 displays the spatial distribution of A_Z_ and A_X_ for the bands which are the most affected by aging (854, 1243, and 1668 cm^−1^) and for one isotropic band (1270 cm^−1^). For each band, the same color scale was applied for both adult and old collagens. Red color corresponds to higher value of A_Z_ or A_X_ than the blue color. The maps generated for amide III band (1270 cm^−1^) showed very low A_Z_ and A_X_ values for both adult and old collagens. This suggests that the orientation of the C-N bonds was not affected by aging. For Amide I band (1668 cm^−1^), the mean value A_Z_ was higher and more homogeneously distributed in old collagen than in adult collagen. This suggests that the C=O bonds are predominantly oriented perpendicularly to the fascicle backbone axis in old collagen (Fig. 6). In addition, A_Z_ values were higher than A_X_ for both adult and old collagens. For the amide III band 1243 cm^−1^, corresponding to parallel C-N bonds, A_X_ was higher than A_Z_ for both adult and old collagens. Concerning the band at 854 cm^−1^ (proline), A_Z_ values were heterogeneously distributed in adult collagen, whereas the distribution of Az values was more homogeneous in old collagen (Fig. 6). Moreover, A_Z_ values were lower in adult than in old collagen. A_X_ values were distributed more homogenously in old collagen when compared to adult collagen. A_X_ values were higher for old than adult collagen.

## Discussion

Density, elasticity and topology of type I collagen have a significant impact on many properties of biological systems. Indeed, aging, fibrosis diabetes and chronic kidney disease are frequently characterized by changes in the organization and mechanical properties of tissues, especially those in which type I collagen is the most abundant. By using PRS, it is possible to provide valuable information at the molecular level in terms of structural organization of type I collagen at a micrometer scale. Selection of specific polarization of incident light and collected Raman signal allows to characterize the preferential orientation of collagen bonds and to identify relevant age-related changes in the structural organization of collagen^22^. In fact, we have used the depolarization ratio and the anisotropic degree of Raman bands to identify the straightness of collagen fibers based on the collagen bond orientation^24^, thereby avoiding potential difficulties of sample preparation and variations of instrumental response^25^.

PRS has been used previously as an original approach to bring information on the chemical composition and structural organization of several matrix components in bone tissues such as mineralized constituents^26,27^, proteoglycans and glycosaminoglycans (GAGs)^28^. This technique permitted also to identify the changes in the orientation of collagen fibers in tendon^15,29^. Masic *et al*. have used PRS approach to study the mechanical behavior of RTTs under uniaxial tensile deformation^16^. Indeed, they have demonstrated the potential of this technique to obtain multiscale information on collagen at molecular level as well as on spatial orientation of collagen fibers. This technique has been also used successfully for cancer diagnosis based on collagen analysis^20,30,31^ and orientation of collagen fibers during skin aging^32,33^. A previous work has also demonstrated the advantage of PRS, when compared to conventional Raman spectroscopy, to better discriminate normal from tumor tissues^34^. Indeed, changes in Raman intensity of collagen bonds were better highlighted using PRS.

In the present study, PRS allowed us to investigate the orientation of collagen fibers with respect to the fascicle backbone axis, and thus the degree of straightness of collagen fibers. RTTs are the appropriate model due to the highly organized collagen fiber network. Thus, this model is widely used to probe the collagen fiber orientation^35^, and to characterize the alterations of biomechanical properties related to aging and diseases^36,37^. Rats develop rapidly during infancy and reach puberty at 2 months of age. In adulthood, each rat month equals approximately 2.5 human years. The age of the old rat has been selected in such a way that it is the period of aging in the animals. 24 months of age in rats corresponds to 60–65 years in Humans^38^. Previous study has accomplished experimental studies in rats and the authors demonstrated a correspondence of 30 days of the man’s life to every day of life of the rat^39^.

Our data demonstrates that the main characteristic bands of type I collagen such as amide I (1668 cm^−1^), Amide III (1243 cm^−1^), hydroxyproline (872 cm^−1^), and proline (854 and 920 cm^−1^) bands are sensitive to light polarization. Maximum intensity of amide I band was obtained with P_ZZ_ polarization because the collagen carbonyl groups are mainly oriented perpendicularly to the fiber axis^16^. The amide III band was composed by two different C-N vibration modes at 1270 cm^−1^ and 1243 cm^−1^, one corresponding to the perpendicular (1270–1300 cm^−1^) and the second corresponding to the parallel position in respect to the collagen fiber axis (1230–1260 cm^−1^)^22^. The intensity of the band at 1270 cm^−1^ was not sensitive to polarization, while the band at 1243 cm^−1^ was not. Accordingly, previous study has shown that when both perpendicular and parallel C–N vibration modes are combined, no changes was observed for this band^22^. However, Janko *et al*. stated that 1246 cm^−1^ was not sensitive to polarization, at the opposite of 1270 cm^−1^ band^40^. Our results show that the band at 1243 cm^−1^ is highly sensitive to polarization (ρ_X_ <0.75). The Raman intensity ratio 1245 cm^−1^/1268 cm^−1^ has been previously used to evaluate i) the orientation of collagen fibers^15^, ii) the degradation and denaturation of collagen^41^, and iii) the disorder in the protein secondary structure^42^. Falgayrac *et al*. have studied the alignment of collagen fibers in cortical bone using specific Raman ratios 1271/ 1243 cm^−1^ and 1668/1243 cm^−1^^17^.

Carotenoids are antioxidants, and substantial *in vitro* work suggests that carotenes are excellent free radical^23^ and can be used as marker substance to characterize the whole antioxidant status of the human epidermis^43,44^. Darvin *et al*. reported that a high antioxidant concentration in human skin could serve as the best protection strategy against premature skin aging^45–47^. Carotenoids have been proposed for the prevention against various chronic diseases^48^. Other optical methods such as resonance Raman spectroscopy, reflection spectroscopy, and skin color measurements could be applied for *in vivo* determination of carotenoids in mammalian skin. Darvin *et al*. reported that the most intense Raman band of carotenoids was localized at 1523–1525 cm^−1^ belonging to the C=C stretching vibration of carotenoids^45^. In our data, this band was observed only in adult collagen with vertical light polarization due to the vertical orientation of long conjugate chain of carotenoids.

We have used pSHG to investigate the age-related alterations in the morphological organization of collagen fibers. Data showed that adult RTTs exhibits fascicles organized as periodic waveform configuration with a long axis parallel to the fascicle backbone axis. However, the fibers become linear and oriented in a parallel manner to backbone axis in old RTTs. This indicated a high straightness of collagen fibers in old RTTs^49^. The comparison between PRS spectra of adult and old RTTs highlighted significant differences, specially for the amide I band (1668 cm^−1^) and the region of 759–938 cm^−1^ related to Proline and Hydroxyproline vibrations. The intensity of the amide I band decreased with age in all polarization modes except P_ZZ_. This could be probably due to the limited sensitivity of the carbonyls groups vibration to polarization by an increase in inter-molecular crosslinks in old collagen which could stabilize fibers^50^. In addition, similar changes in relative intensity of Proline and Hydroxyproline vibrations (939, 920, 875, 854 cm^−1^) were observed between adult and old RTTs. Indeed, the bonds corresponding to these vibrations are oriented nearly in parallel than perpendicular direction to the fascicle backbone axis.

Due to the difference in collagen fibers orientation between adult (periodic waveform) and old RTTs (aligned to the fascicle backbone axis), we used PRS mapping mode to take into account the spectral variability in analyzed samples. A previous work has reported the importance of such strategy to investigate the changes in the orientation of collagen fibers in bone tissue^51^ and to obtain 3D structural information on collagen from a highly complex biological systems^24,52^. We then evaluated the distribution of the anisotropy degree using three anisotropic bands affected by aging (854, 1243, and 1668 cm^−1^) and one isotropic vibration (1270 cm^−1^). Indeed, this anisotropic mapping was previously used to detect early dental caries^25^. Our data demonstrates that the anisotropy degree allow the accurate monitoring of the variability in the orientation of collagen fibers with aging. It is important to note that we have performed all PRS measurements on hydrated RTTs. In fact, water plays a crucial role in the stabilization of collagen triple helical conformation and fiber assembly^53^. Dehydration is then considered as a factor responsible for alterations in type I collagen structural organization and mechanical properties^54–56^.

Aging is able to induce physical alterations in the matrix proteins of tissues. These changes, which are initiated by biochemical processes, have direct consequences on molecular and structural organization of these proteins, especially of the most abundant component, type I collagen. These molecular alterations at a nano-scale level induce at macroscopic level mechanical alterations that could affect several tissue functions. Thus, by altering the mechanical properties or matrix proteins such type I collagen (stiffness, length and diameter of type I collagen …), aging could for example affect elastic properties of many tissues (skin, cardiovascular endothelium, connective tissues…). These alterations are the largest risk factor in the development of many diseases, which can become pathological, causing thereby co-morbidities, need to be characterized a molecular level to better understand the role of such changes in the gradual loss of autonomy in the elderly^57^. Therefore, interventions that delay ageing or age‐related diseases would greatly benefit health. Rats model is generally used to mimic human conditions and to support the hypothesis of research.

## Conclusion

This study demonstrates de potential of PRS combined to pSHG technique to provide precious information regarding the changes in morphological and structural organization of type I collagen during chronological aging. We have first characterized the sensitivity of Raman bands to polarization and their anisotropy. We have correlated these properties to the privileged orientations of the chemical bonds of collagen with respect to the fiber axis.

For a higher degree of fiber alignment to the fascicle backbone axis, the depolarization ratio of certain collagen bands was lower and the anisotropy degree was higher. We have investigated the age-related changes in the orientation of collagen fibers. We did not observe significant differences in the spectral region 1000–1500 cm^−1^ between adult and old RTTs. However, the anisotropy of the other bands was higher in old RTTs, indicating a higher straightness of collagen fibers. These data suggest that the most age-related changes in the orientation of collagen fibers occur with laser polarization perpendicular to the fascicle backbone axis. Finally, PRS can be used to provide further insights into the relationship between structural organization of collagen and biomechanical properties of tissues.

## Materials and Methods

### Rat tail tendon

The animal procedure was approved by the local ethics committee in animal experimentation of Reims Champagne-Ardenne (C2EA, registration 56, France) and the experiments were performed in accordance with European directive 2010/63/UE. Rat tail tendons (RTTs) specimens were obtained from adult (2 months, n = 6) and old (24 months, n = 6) Wistar rats. The animals were anesthetized with isoflurane and sacrificed. Tails were detached and then stored at −80 °C until further use. At day experiments, the skin was removed from the tails exposing all tendons, skeletal frame, and vascular system. To prevent tensile loading during tendon extraction, a flat clamp was used to hold the distal part of the tails. The vertebrae located in the tail were gradually broken with circular movement with the clamp in order to release the tendon from the skeletal structure and the vascular system. Tendon fascicles were then lifted and washed with physiological buffer at pH = 7.4. Two fascicle pieces of approximatively 50 mm in length were cut from each sample. For Raman measurements, one hydrated fascicle piece was placed on CaF2 slides in quartz Petri dishes of PBS. The samples were kept totally immersed in PBS and oriented according to the x-axis direction during PRS and conventional Raman acquisitions, to prevent their dehydration. The other piece was placed on 35 mm cover-glass Petri dish for pSHG analysis.

### pSHG acquisition method

pSHG images were collected with a Zeiss LSM 710-NLO microscope (Zeiss Microsystems, Marly le Roi, France) using 20X objective (NA 0.8). Laser excitation at 860 nm was provided by a CHAMELEON femtosecond Titanium-Sapphire laser (Coherent, Courtaboeuf, France). Laser power on the sample was adjusted up to 20 mW. RTTs samples were oriented according to the fibers long axis in the horizontal direction corresponding to the x-polarization. Backward pSHG images (425 µm × 425 µm) were collected from RTTs with a 420–440 nm bandpass filter using ZEN imaging software.

### Polarized Raman spectrometer

**Figure 7:**
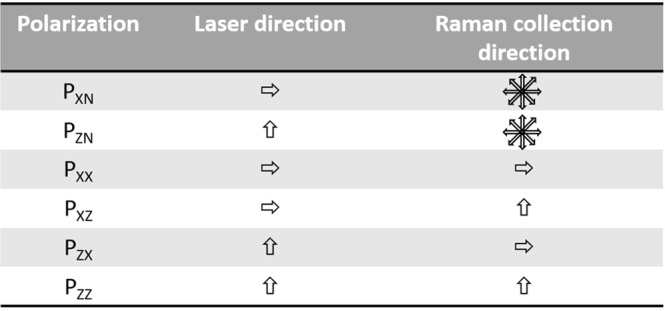
Different Combinations between polarization directions of laser excitation and collected Raman scattering.

Raman spectra were recorded with a near infrared confocal Raman spectrometer (Labram ARAMIS, Horiba Jobin Yvon S.A.S., France). This setup consisted of a microscope (Olympus, BX41, France) coupled to the Raman spectrometer equipped with 600 groove/mm diffraction grating. The microscope was equipped with a xy-motorized (Marzhauser, Germany), computer controlled sample stage, which enabled automatic scanning of the sample with a spatial resolution of 1 μm. The excitation source (785 nm) was provided by diode laser (Toptica Photonics, Germany) delivering 60 mW of laser power on the sample. This laser beam was focused on the sample with water immersion NIR 100x objective (NA 1.0, Olympus, France). The backscattered light was collected by the objective and transmitted to the spectrometer equipped with a Pelletier-cooled charge-coupled device (CCD) detector. The incident laser was initially polarized horizontally (X direction). A linear polarizer was placed into the laser path to select Z direction (perpendicular) laser polarization. Another linear analyzer was placed before the spectrograph to select parallel and perpendicular scattered light components with respect to the polarization of incident light. A scrambler was positioned before the 600 groove/mm holographic grating in order to minimize the polarization-dependent response of the spectrograph. Polarized Raman maps were performed on several hydrated adult (n = 6) and old (n = 6) RTTs by combining different polarizations of incident and collected Raman scattering (Fig. 7). From each tendon, 3–4 randomly selected fascicles were analyzed by polarized Raman microspectroscopy. Non-polarized (P_XN_ and P_ZN_) and polarized Raman (P_XX_, P_ZZ_, P_XZ_, and P_ZX_) maps **(**900 spectra**)** were recorded on the same areas on each fascicle with spatial resolution of 1.5 µm to take in account the intra- and inter- fascicles variability with age. Each spectrum was acquired using 20 seconds integration time in the 600–1800 cm^−1^ spectral region with a spectral resolution of 4 cm^−1^. P_XN_ and P_ZN_ were considered as unpolarized data because the linear analyzer was removed to collect all scattered light. Data acquisition was carried out by means of the LabSpec 5 software (Horiba Jobin Yvon S.A.S. France).

### Data pretreatment and treatment

After acquisition, spectra were first calibrated using Raman calibration standards^58^. The spectrum of the halogen lamp was used to correct for the wavelength-dependent signal detection efficiency of the Raman setup. All spectra were Caf_2_ interference subtracted, baseline corrected using a fourth order polynomial and smoothed with fifth points Savitzky-Golay algorithm in order to minimize the influence of noises. The resulting spectra were then normalized to the band at 1450 cm^−1^ related to CH_2_/CH_3_ deformations. In fact, Janko and al., reported that this isotropic band doesn’t shows a preferential orientation and was equally distributed across the collagen amino acid sequence^40^.

pSHG images were analyzed using Open source NIH ImageJ software (Wayne Rasband, National Institutes of Health, Bethesda, MD) to determine the mean diameters of adult and old fascicles and fiber bundles. Perpendicular lines to RTTs long axis were drawn on several RTTs samples to detect the fascicles and fiber bundles periodicity. Two-dimensional plot profiles were then obtained displaying the intensities of pixels (y –axis) along a line (x-axis) within the adult and old pSHG images. The diameter of fascicles and fiber bundles were measured as the mean width of periodic waveforms.

### Deconvolution procedure

Raman spectra of collagen show a complex set of overlapping bands. In order to identify a number of sub-bands within a collagen spectral region, peak deconvolution procedure was applied on whole spectral region using a mixed Gaussian and Lorentzian peak shape. Peaks constituting the spectrum and their full width at half-maximum were manually selected in order to define the starting conditions for the best-fit procedure. The best fit procedure was then performed to determine convolution peaks with optimized intensity, position and width. The quality of the fit was estimated by the standard error and the χ^2^ values. Integrated intensity of each individual polarized Raman subband of collagen was estimated. The depolarization ratios and anisotropy degree of collagen bands were calculated and used to monitor the molecular and structural changes of type I collagen with aging.

### Depolarization ratios and anisotropy degree

Depolarization ratio (ρ) is defined as the ratio of integrated peak intensities of perpendicular to parallel Raman scattered light intensities ($\frac{I\perp }{I\Vert }$) with respect to the polarization direction of the incident laser beam. ρ was used to calculate the anisotropic responses of all collagen bands in order to identify those bands that are sensitive to polarization directions. When ρ is less than 0.75, a band was considered as polarized, otherwise it was referred as depolarized^59^. Two different depolarization ratios I_ZX_/I_ZZ_ (ρ_z_) and I_XZ_/I_XX_ (ρ_x_) were calculated on each collagen band from adult and old RTTs. Depolarization ratios were then plotted as function of Raman shift in order to investigate the structural changes in collagen organization and orientation according to the age. The standard error was estimated by dividing the standard deviation calculated on depolarization ratios on each adult and old collagen band by the square root of the sample size^60^.

Polarization anisotropy degree (A) was used to estimate the degree and the direction of collagen fibers alignment in adult and old RTTs. Two Polarization anisotropic degrees (A_X_ and A_Z_) were performed on adult and old collagen bands as function of polarization of laser direction according the following formula:

$${{\rm{A}}}_{{\rm{X}}}=\frac{IXX-IXZ}{IXX+2\ast IXZ}\,\,\,\,\,\,\,{{\rm{A}}}_{{\rm{Z}}}=\frac{IZZ-IZX}{IZZ+2\ast IZX}$$

A depolarization ratios and anisotropic degree maps were generated in order to investigate the difference in the structural organization and orientation, and alignment of collagen fibers with chronological aging^25^. Analysis of Variance (ANOVA) test was performed on these ratios to find statistical significant differences between collagen bands from adult and old RTTs samples (*p < 0.05, **p < 0.01, ***p < 0.001).

## Data Availability

The datasets generated during and/or analyzed during the current study are available from the corresponding author on reasonable request.
